# Alzheimer’s disease and memantine effects on NMDA-receptor blockade: non-invasive in vivo insights from magnetoencephalography

**DOI:** 10.1038/s41380-025-03288-3

**Published:** 2025-10-10

**Authors:** Juliette H. Lanskey, Amirhossein Jafarian, Laura E. Hughes, Melek Karadag, Ece Kocagoncu, Matthew A. Rouse, Natalie E. Adams, Michelle Naessens, Vanessa Raymont, Mark Woolrich, Krish D. Singh, Richard N. Henson, James B. Rowe

**Affiliations:** 1 MRC Cognition and Brain Sciences Unit, University of Cambridge, Cambridge, UK; 2 Department of Clinical Neurosciences and Cambridge University Hospitals NHS Foundation Trust, Cambridge Biomedical Campus, Cambridge, UK; 3 Department of Psychiatry, University of Oxford, Oxford, UK; 4 Oxford Centre for Human Brain Activity, Wellcome Centre for Integrative Neuroimaging, Department of Psychiatry, University of Oxford, Oxford, UK; 5 Cardiff University Brain Research Imaging Centre, School of Psychology, Cardiff University, Cardiff, UK; 6 Department of Psychiatry, University of Cambridge, Cambridge, UK

**Keywords:** Physiology, Diseases, Biomarkers, Cell biology, Neuroscience

## Abstract

To accelerate new treatments for Alzheimer’s disease, there is the need for human pathophysiological biomarkers that are sensitive to treatment and disease mechanisms. In this proof-of-concept study, we assess new biophysical models of non-invasive human MEG imaging to test the pharmacological and disease modulation of NMDA-receptor inhibition. Magnetoencephalography was recorded during an auditory mismatch negativity paradigm from (1) neurologically-healthy people on memantine or placebo (n = 19, placebo-controlled crossover design); (2) people with Alzheimer’s disease at baseline and 16-months (n = 42, amyloid-biomarker positive, longitudinal observational design). Optimised dynamic causal models inferred voltage-dependent NMDA-receptor blockade using Parametric Empirical Bayes to test group effects. The mismatch negativity amplitude was attenuated when Alzheimer’s disease was more severe (lower baseline mini-mental state examination) and after follow-up (*versus* baseline). Memantine increased NMDA-receptor inhibition, compared to placebo. Alzheimer’s disease reduced NMDA-receptor inhibition in proportion to severity and over time. In line with preclinical studies, we confirm in humans that memantine and Alzheimer’s disease have opposing effects on NMDA-receptor inhibition. The ability to infer such receptor dynamics and pharmacology from non-invasive physiological recordings has wide applications, including the assessment of other neurological disorders and novel drugs intended for symptomatic or disease-modifying treatments.

## Introduction

Early in the pathogenesis of Alzheimer’s disease, there is impairment and loss of synapses [1]. Synaptic density is closely related to cognitive impairment [2] and the maintenance and restoration of synaptic health is an area of strong therapeutic interest [3]. Voltage-dependent NMDA type glutamatergic receptors are critical to synaptic function, plasticity for memory and are implicated in the pathogenesis of Alzheimer’s disease. NMDA-receptors are subject to voltage-dependent blockade by magnesium ions [4]. This magnesium inhibition of NMDA-receptors is reduced in Alzheimer’s disease, with reduced magnesium ion occupation of the NMDA-receptor channel pores even at low levels of depolarisation [5, 6]. This leads to over-activation of NMDA receptors, with excess calcium influx disruptive to cell function and cognition [7]. Since 2004, the drug memantine has been licenced to treat moderate to severe Alzheimer’s disease [8, 9]. Memantine blocks NMDA-receptor channels when they are pathologically open at low-levels of depolarisation, without affecting the neurotransmission when the post-synaptic membrane is sufficiently depolarised [5].

NMDA-channel kinetics [10] and the impact of Alzheimer’s disease on the voltage dependency of NMDA receptors [11] have been studied extensively in tissue cultures [12], animal studies [13] and *post mortem* data [14–16]. These previous studies provide a strong foundation for the development of biophysical models suitable for use in early-phase trials for people with, or at risk of, Alzheimer’s disease. In particular, a technique called dynamic causal modelling can be exploited to reveal disease-effects and therapeutic mechanisms non-invasively in humans to the level of cell-classes and neurotransmitters [17–20]. Dynamic causal modelling shows high reliability over odd and even trials [18] and over test-retest sessions [21] and identifies cellular mechanisms underlying evoked responses, such as magneto- and electro- encephalographic observations [22, 23]. The generation of evoked responses to unexpected stimuli, relies on intact NMDA transmission within and between nodes in neural information processing hierarchies [24]. This effect is dose-dependently blocked by NMDA-receptor blockers [25].

NMDA receptor dysfunction contributes to synaptic transmission deficits in Alzheimer’s disease [26]. In Alzheimer’s disease, it is proposed that increased intracellular calcium at rest (due to NMDA receptor dysfunction) [15] increases background calcium ion ‘noise’ leading to impaired synaptic signal detection [11]. Indeed, the evoked mismatch negativity response amplitude is reduced in Alzheimer’s disease [27]. This creates the opportunity to model in vivo the generators of evoked responses, and validate the dynamic causal modelling approach. Specifically, one can establish validity of the models, via the effects of drugs, like memantine, that act on NMDA-receptors and the effects of Alzheimer’s disease on cortical generators.

In this paper, we aim to assess the suitability of dynamic causal modelling to support clinical trials by (1) identifying therapeutic target engagement and (2) measuring the respective target’s importance to cognitive decline and progression of Alzheimer’s disease. To do this, we first identify the target of memantine in humans, in vivo, using dynamic causal modelling. We then measure how the same model parameter relates to cognitive decline and disease progression in people with symptomatic Alzheimer’s disease (beta-amyloid biomarker positive, with amnestic mild cognitive impairment or early dementia). In doing so, we confirm sensitivity of magnetoencephalography (MEG) and dynamic causal modelling to both the severity and progression of Alzheimer’s disease. Importantly, we demonstrate that dementia and memantine treatment have opposing effects on the cortical microcircuit. We use data from two separate studies: (1) a randomised placebo-controlled double-blinded crossover study of memantine in healthy controls; and (2) a longitudinal study of people with Alzheimer’s disease. We tested the following hypotheses: 1a) the mechanism of action of memantine is blockade of NMDA-receptors; 1b) memantine increases the blockade of NMDA channels; 2a) the mismatch negativity amplitude is reduced in Alzheimer’s disease, more so with disease severity and progression; 2b) NMDA blockade inferred from dynamic causal models (DCMs) is lower in people with more severe Alzheimer’s disease (e.g. lower mini-mental state examination, MMSE) and 2c) progression of Alzheimer’s disease reduces the inferred blockade of NMDA channels (baseline *versus* follow up).

## Results

### Effects of memantine and alzheimer’s disease on the mismatch negativity

**Fig. 1:**
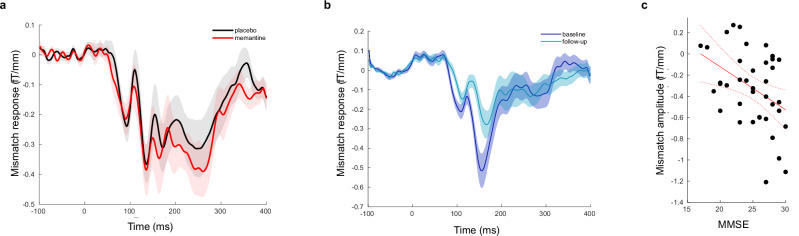
Mismatch negativity waveform. **a** The mismatch negativity waveform on memantine and placebo in control participants from the memantine-placebo study. There was no significant difference between sessions. **b** The mismatch negativity waveform for people with Alzheimer’s disease from the NTAD study at baseline and follow up. The amplitude was significantly reduced over time by Alzheimer’s disease (baseline *versus* follow-up for people with Alzheimer’s disease from the NTAD study, t = −2.92, p = 0.003). **c** The mismatch negativity amplitude correlated with MMSE for people with Alzheimer’s disease from the NTAD study (r = −0.4, p = 0.01).

Mismatch negativity waveforms were calculated as the difference between responses to deviant and repeated tones. T-tests assessed differences in the average mismatch negativity amplitude over the a priori interval 140–160 ms [28]. Compared to placebo, memantine did not significantly alter the mismatch negativity response between 140–160 ms (Fig. 1a). Note that whereas there was no difference in the average around the *peak* of the mismatch negativity amplitude (i.e. 140–160 ms), the dynamic causal modelling considers the entire waveform over all timepoints from 0–300 ms.

For participants with Alzheimer’s disease from the New Therapeutics in Alzheimer’s disease (NTAD) study, the mismatch negativity was significantly reduced, compared to controls (t = −2.56, p = 0.007, see supplementary materials).The mismatch negativity response was further reduced over time in people with Alzheimer’s disease (baseline *versus* 16-month follow up, t = 2.92, p = −0.003, Fig. 1b) with a medium effect size (d = 0.60). For participants with Alzheimer’s disease, the mismatch negativity amplitude correlated with MMSE; people with lower MMSE (and hence likely to have more severe Alzheimer’s disease) had a smaller mismatch negativity amplitude (r = −0.4, p = 0.01, Fig. 1c).

### The generative model of the mismatch negativity response

**Fig. 2:**
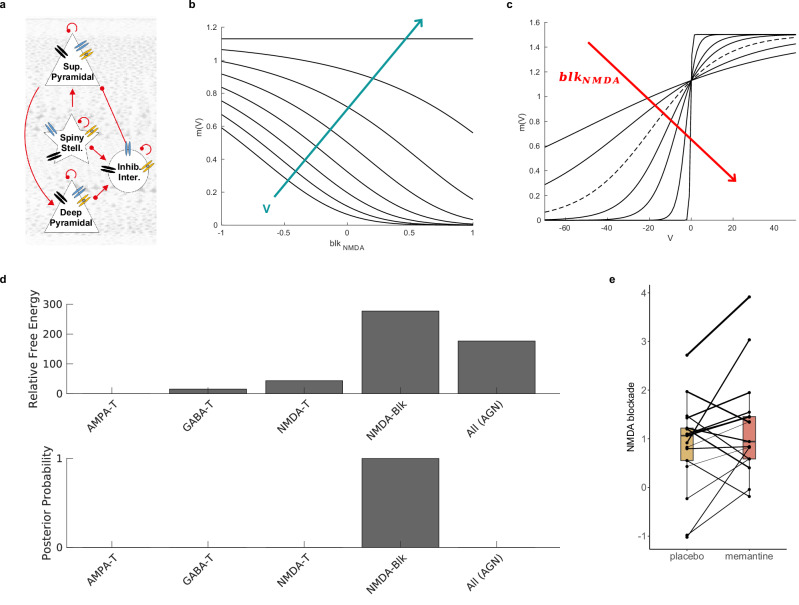
The generative canonical microcircuit conductance model with NMDA channel blockade parameters and the mechanism of memantine. **a** The intrinsic connectivity between cell populations within each region of the model. The NMDA switch function (Eq. 2) plotted (**b**) against the NMDA blockade parameter shown for increasing voltage values (from −70–0 V in 10 V steps) and (**c**) against voltage shown for increasing values of the exponential of the NMDA blockade parameter ${{{\boldsymbol{blk}}}}_{{{\boldsymbol{NMDA}}}}$ (−1, −0.5, 0, 0.5, 1, 2 and 4). The dashed line shows the NMDA switch function with the blockade parameter value set at the default value from the original model [82]. As the blockade parameter increases, the magnesium switch function output, which scales NMDA channel conductance, reduces. **d** Free energy and posterior probabilities of PEB models explaining the effect of memantine versus placebo. The PEB analysis with the NMDA channel blockade parameters had the highest posterior probability for explaining differences between neurophysiological mismatch negativity responses on placebo versus drug; memantine acts primarily on the NMDA blockade parameter. **e** Memantine increases the NMDA channel blockade parameter with a posterior probability >95% in the left parietal cortex. Lines are weighted by each subject’s average precision of NMDA blockade over sessions. Sup., superficial; stell., stellate; inter., interneuron; m(V), the switch function output; blk_NMDA_, the NMDA blockade parameter; V, voltage; AMPA-T, AMPA channel time constant; GABA-T, GABA channel time constant; NMDA-T; NMDA channel time constant; NMDA-Blk, NMDA channel blockade; All (AGN); AMPA, GABA and NMDA time constants and NMDA blockade parameters.

Dynamic causal modelling is a standard translational modelling approach which uses variational Bayesian inference to infer synaptic physiology and model evidence from neuroimaging data. Here, we modified a conductance-based canonical microcircuit DCM to allow inference of subject-specific NMDA channel blockade from evoked MEG responses (Fig. 2a, see methods section for full details). In brief, we defined the prior distribution of the NMDA channel blockade parameter (blk_NMDA_) as a normal distribution ${bl}{k}_{{nmda}} \sim N\left(m,\sigma \right)$, with prior mean of ${{\rm{m}}}=0$ and variance of $\sigma =\frac{1}{64}$ (in line with the default variance of other channel time constants in such models). The NMDA blockade parameter is exponentially transformed to assure positivity constraints:

1 $${\alpha }_{{NMDA}}=\exp ({{blk}}_{{NMDA}})$$

The ${\alpha }_{{nmda}}$ parameter is scaled by its default physiological value (i.e., $-0.06$) and modulates the membrane potential $V$ through a sigmoid transformation (Fig. 2b) [29]:

2 $$m\left(V\right)=\frac{1.50265}{1+0.33\exp (-0.06{\alpha }_{{NMDA}}V)}$$

The m(V) modulates the membrane potential in the DCM (please see Methods section). A consequence of this model is that the higher the NMDA blockade parameter, the less conductance is mediated by the NMDA channel (Fig. 2c).

### Increased blockade of NMDA channels explains the memantine effect

Previous application of DCM to the mismatch negativity has used a bilateral network of brain regions. While some studies have focussed on the frontotemporal network generators of the mismatch negativity [30–34], including auditory, superior temporal, inferior frontal cortex, others have also included parietal cortices [27, 35–37]. The parietal cortex is also involved in mismatch negativity generation [38–42] and has high sensitivity to Alzheimer’s disease [43]. Here we focus on just parietal cortex, given its involvement in Alzheimer’s disease and mismatch negativity generation [39, 40], to reduce model complexity [22]. The model was fitted first to the control participants from the memantine-placebo study. Parametric Empirical Bayes (PEB) [44] was used to compare 5 models, in which memantine affected the NMDA channel blockade parameter (blk_NMDA_), or the AMPA, GABA or NMDA time constants or a fifth model in which it affected all of these. The posterior probability was highest for the blk_NMDA_ model (indeed, close to 1), demonstrating that the effect of memantine was most parsimoniously explained by changes in NMDA channel blockade (Fig. 2d). Memantine increased NMDA channel blockade with a meaningful effect (probability of parameter >95%) in the left parietal cortex (posterior estimate = 0.41, posterior probability = 1). PEB posterior estimates of the expected NMDA blockade value were extracted for each participant at each session and plotted (see Fig. 2e). Bayesian model comparison and averaging [44] also showed that the NMDA channel blockade parameter was greater on memantine than placebo (posterior estimate = 0.42, posterior probability = 1).

### Effect of severity and progression of alzheimer’s disease

**Fig. 3:**
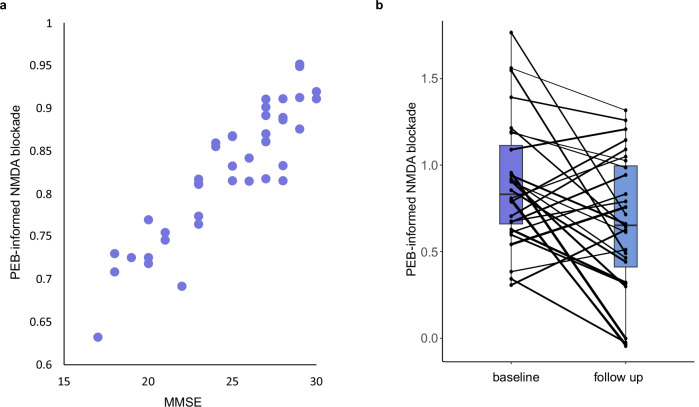
NMDA channel blockade is affected by Alzheimer’s disease in right parietal cortex. The PEB posterior estimate of the expected value of the NMDA channel blockade parameter for each person with Alzheimer’s disease are shown. This NMDA-blockade parameter is re-estimated during the second-level PEB analysis. NMDA channel blockade (**a**) correlates with MMSE (posterior estimate of the second-level NMDA channel blockade parameter=0.06, posterior probability = 0.99) and (**b**) reduces further at follow-up compared to baseline (posterior estimate of the second-level NMDA blockade parameter = −0.125, posterior probability = 0.97). Lines are weighted by each subject’s average precision of NMDA blockade over sessions. MMSE mini-mental state examination, PEB parametric empirical Bayes.

For the Alzheimer’s disease group, PEB analyses were conducted to identify (1) the effect of disease severity (MMSE score) on NMDA channel blockade and (2) the effect of disease progression on NMDA channel blockade (baseline *versus* follow-up). NMDA channel blockade was reduced with more severe Alzheimer’s disease (a lower MMSE score) with a meaningful effect (posterior probability of parameter > 0.95) in right parietal cortex (posterior estimate = 0.06, posterior probability = 0.99). PEB posterior estimates of expected NMDA channel blockade values were extracted from each subject’s model after application of PEB. The relationship with MMSE is illustrated in Fig. 3a). Bayesian model comparison and averaging also showed that NMDA channel blockade of the right parietal cortex was reduced with more severe Alzheimer’s disease (posterior estimate = 0.05, posterior probability = 0.79). The NMDA channel blockade further reduced at follow-up compared to baseline in the right parietal cortex (posterior estimate = −0.125, posterior probability=0.97). Expected values were extracted from the PEB and plotted (Fig. 3b). Bayesian model comparison and averaging also showed that NMDA channel blockade further reduced at follow-up compared to baseline in the right parietal cortex (posterior estimate = −0.08, posterior probability = 0.68).

## Discussion

There are three principal results of this study: (i) by inversion of MEG data to a biophysically informed generative model, we confirmed that memantine increases the NMDA blockade parameter; (ii) Alzheimer’s disease severity is associated with the opposite effect on NMDA blockade; and (iii) Alzheimer’s disease progression further reduces the blockade, within subject. The effect of drug and disease on NMDA receptor blockade may not in itself be surprising; what is important is that this inference can be made from non-invasive human imaging data using a relatively simple dynamic causal model. As the NMDA blockade parameter changes, conductance through NMDA channels becomes increasingly non-linear, requiring more depolarisation for channel conductance, and this can explain why the mismatch negativity amplitude attenuates with disease severity and with disease progression over 16 months. We have shown that this approach to non-invasive neurophysiological data acquired in vivo is feasible as a foundation to experimental medicine studies in people with Alzheimer’s disease.

To set these disease-specific results in context, clinical trials have lower costs and attrition when based on stronger target validation [45]. Experimental medicines studies can be used to demonstrate pharmacological target *engagement* and target *relevance* to a disorder [46]. Target engagement for organs other than the brain can often be measured directly, but the blood-brain barrier, skull enclosure, and lack of regenerative capacity make direct brain assays unrealistic. Neurochemical imaging by positron emission tomography and single photon emission computerised tomography with selective tracers are possible for some molecular targets, e.g. for the measurement of dopamine receptor occupancy by dopamine agonists [47]. An alternative approach using biophysically informed DCMs has been used to study mechanisms of action of ketamine [48], galantamine [28, 49] and tiagabine [34, 50], as well as natural experiments afforded by anti-NMDA antibody mediated encephalitis and inherited channelopathies [51, 52].

Here, we confirmed that memantine increases the blockade of NMDA channels. The original direct assays of memantine’s blockade of NMDA receptors included patch-clamp recordings [53] and *post* *mortem* tissue [54]. Rodent models with PET imaging confirmed NMDA receptor blockade by a memantine derivative [55]. In silico studies suggested that memantine may have a neuroprotective effect on Alzheimer’s disease [56], even if current licenced applications are symptomatic rather than disease modifying in their intention. Nonetheless, chronic in vivo clinical use of memantine by people with Alzheimer’s disease increases cortical metabolism in temporal and parietal regions [57] along with clinical benefit [9]. Our study was not designed or powered to show clinical efficacy; rather its aim was to confirm the mechanism of action, as proof of concept for the dynamic causal modelling methodology.

Age has previously been associated with reduced NMDA receptor function [58]. Indeed, we found that age increased the block of NMDA receptors (i.e. reduced NMDA receptor function, see supplementary materials). This reduced NMDA receptor function with advancing age may be exacerbated in Alzheimer’s disease [59]. Compared to control participants, we found that people with Alzheimer’s disease had increased functional NMDA receptor blockade (i.e. reduced NMDA receptor function, see supplementary analysis). This is consistent with previous findings [60] from animal-models of Alzheimer’s disease [61] and human *post- mortem* studies [15, 62]. This is also in line with evidence that therapeutically enhancing NMDA receptor function is beneficial for people with early-stage Alzheimer’s disease [63, 64]. Yet, as we show, Alzheimer’s disease severity and progression *increase* relative NMDA receptor function [11, 65, 66]. This apparent contradiction is addressed by Olney and colleagues, who proposed that a disinhibition state triggered by NMDA receptor hypoactivity leads to low-grade chronic excitotoxic activity, exacerbating neuronal degeneration [59]. Indeed, studies have shown that in response to reduced NMDA receptor function, a typical consequence is excessive glutamate release [67–69].

In Alzheimer’s disease, this low but chronic influx of calcium through pathologically-open NMDA receptors may potentiate excitotoxicity [70] and cell death [71]. Although it was theorised that memantine would therefore delay cell death [7], by blocking excitotoxic calcium entry, memantine remains in use as a symptomatic treatment. Note too that memantine is only licenced for moderate to severe Alzheimer’s disease (approximately MMSE < 20) [9, 72], reflecting a complex and dynamic evolution of the role NMDA-receptor function in Alzheimer’s disease.

An advantage of dynamic causal modelling of evoked neurophysiological responses is the ability to bridge between clinical and preclinical models of disease. It can inform the understanding of the biological processes that generate the neurophysiological responses underlying cognitive task performance. Here, we found that the mismatch negativity amplitude is significantly correlated with MMSE and significantly reduces with disease progression. This is an important demonstration in its own right, given the need for quantitative biological tools to enhance early-phase clinical trials. However, the greater value of this study is in the analysis of disease and drug mechanism in vivo. Specifically, how NMDA channel blockade relates to the neurophysiological deficit and cognitive decline.

A progressive reduction in mismatch negativity amplitude in people with Alzheimer’s disease compared to controls has been shown previously in Alzheimer’s disease [27].Other studies have shown that the mismatch negativity is significantly associated with verbal learning [73], self-reported disability [73], cognitive training [74] and episodic memory [75] in people with mild cognitive impairment [73, 75] and Alzheimer’s disease [74]. The current task (a roving mismatch negativity paradigm) is sensitive to disease presence, as shown previously [27], and disease severity and progression, which is especially encouraging given the ease of the task for participants and the robustness of the mismatch negativity waveform [76]. The mismatch negativity is reduced in schizophrenia, which is also characterised by NMDA receptor dysfunction [77], and negatively correlates with symptom severity [78]. This suggests the measure is specific not just to Alzheimer’s disease but also to other diseases that affect the cortical generators of the mismatch negativity. Future studies could employ the same methodology to assess target engagement in people with other disorders affecting NMDA-receptor blockade.

There are several limitations to our study. First, though the models were informed by human disease, there is no complete model of the disease [17]. Second, we only modelled two brain regions. This was to reduce model complexity [22], although we recognise that the mismatch negativity is generated by a wider network [27, 31, 33, 37]. Fourth, we recruited according to clinical diagnoses of Alzheimer’s disease, and mild cognitive impairment. However, all patient participants were positive for amyloid biomarkers, by cerebrospinal fluid examination or positron emission tomography. Finally, the longitudinal study attracted an attrition rate of 29% over a mean interval of 16 months. This was similar to protocol expectation (20% per annum), but the longer than planned interval reflects the impact of the COVID-19 pandemic. This may have biased results to the remaining sample.

In conclusion, the dynamic causal modelling approach enabled non-invasive assessment of NMDA-receptor blockade in humans, in vivo. Data were recorded during a robust paradigm, for the mismatch negativity response, which is sensitive to Alzheimer’s disease, disease severity and progression. The biologically-informed generative models reproduced the neurophysiological deficit and indicated appropriate drug target engagement: exemplified by increased NMDA receptor blockade by memantine. The study demonstrates target engagement and target relevance. Future translational studies could tailor generative models to measure other drugs and targets of interest, as part of early phase clinical trials of much needed novel dementia therapeutics.

## Methods

The study has two principal parts. The first part is the analysis of a randomised, placebo-controlled double-blind crossover study with healthy adults [79]. The second part is a longitudinal study of people with Alzheimer’s disease, including its prodromal state of mild cognitive impairment [80].

### Participants

For the placebo-memantine crossover study [79], 19 neurologically healthy people completed two MEG sessions, two weeks apart where they received either (1) placebo or (2) 10 mg oral memantine. Written informed consent was acquired in accordance with the Declaration of Helsinki (1991) from all participants. The study was approved by the local ethics committee and exempted from Clinical Trials status by the UK Medicines and Healthcare products Regulatory Agency. The International Standard Randomised Controlled Trial Number is 10616794. Power analyses were conducted and reported previously [79]. The MEG scan was conducted three hours after drug administration, in line with estimated peak concentration. See Table 1 for participant demographics.

**Table 1:** Memantine-placebo control participant demographics.

Sex (Male:Female)	Handedness (Right:Left:Both)	Age (yrs)	Education (yrs)	Baseline MMSE
14:5	19:0:0	67.1 (±7.29)	15.5 (±3.27)	29.6 (±0.50)

*Yrs* years, *MMSE* mini-mental state examination.

From the NTAD study[80], we include MRI, MEG and cognitive data from people with amyloid-positive mild cognitive impairment or Alzheimer’s disease dementia (n = 50). Written informed consent was acquired in accordance with the Declaration of Helsinki (1991) from all participants. The study was approved by the local ethics committee, the East of England Cambridge Central Research Ethics Committee (REC reference 18/EE/0042). Power analyses were conducted and reported previously [80]. Two people were excluded who did not complete the mismatch negativity paradigm because the earphones did not fit comfortably, two people whose diagnosis was revised during follow-up and one person due to data recording technical issues. Three people with Alzheimer’s disease were taking memantine as prescribed and excluded from the analysis (final n = 42, see Table 2 for participant demographics). We also include MRI, MEG and cognitive data from 30 of the participants with mild cognitive impairment or Alzheimer’s disease who completed a follow-up scan at an average of 16 months after the baseline MEG scan.

**Table 2:** NTAD patient participant demographics at screening.

Sex (M:F)	Handedness (R:L:B)	Age (yrs)	Education (yrs)	Baseline MMSE	Baseline PET (SUVR)	Baseline CSF (tau/A-beta 1–42)
18:24	37:4:1	73.6(±7.37)	14.2 (±3.94)	24.9 (±3.61)	1.66 (±0.18)	2.19 (±1.28)

*M* male, *F* female, *R* right, *L* left, *B* ambidextrous, *MMSE* mini-mental state examination, *PET* positron emission tomography, *SUVR* standardized uptake value ratio, *CSF* cerebrospinal fluid, *Yrs* years, *MMSE* mini-mental state examination.

### Data collection

For the memantine-placebo study [79], MEG data were recorded three hours after drug or placebo administration while participants listened passively to a 3 × 5 min roving mismatch negativity paradigm. Tones repeated at frequencies of 400–800 Hz with 75 ms duration at 500 ms intervals. After 3–10 repetitions, the tone frequency changed pseudorandomly (with an approximate Poisson distribution). MEG was recorded with the Elekta VectorView system, configured with 204 planar gradiometers and 102 magnetometers at 102 locations. Eye movements and head position were measured with vertical and horizontal electrooculography and 5 head position indicator coils, respectively. Nasion and pre-auricular fiducial points were measured with a 3D digitizer (Fastrak Polhemus Inc., Colchester, VA), with over 60 additional scalp surface points. T1-weighted MRI was recorded with a 7 T Siemens TERRA scanner using a magnetisation prepared 2 rapid gradient echo (MP2RAGE) sequence.

For the NTAD study [80], MEG data were recorded while participants listened passively to a 2 × 5 min roving mismatch negativity paradigm. Tones repeated at frequencies of 400–800 Hz varying in 50 Hz steps with 75 ms duration at 500 ms intervals. After 3–10 repetitions, the tone frequency changed. MEG was recorded with the Elekta VectorView system and MEGIN Triux Neo scanner, with 204 planar gradiometers and 102 magnetometers at 102 locations. We recorded electrocardiogram data with 2 electrodes on the right clavicle and left, lower rib; electrooculography data with an electrode below and above the left eye and on bilateral canthi; and head position with five head position indicator coils, standard fiducial points and over 500 additional scalp surface points with a 3D digitizer (Fastrak Polhemus Inc., Colchester, VA). T1-weighted MRI was recorded with a 3 T Siemens PRISMA scanner using a magnetisation prepared rapid gradient echo (MPRAGE) sequence.

### Preprocessing

Preprocessing of data from both studies followed the same pipeline as previously reported [27]. In brief, MaxFilter v2.2 software was used. Independent component analysis of data using the EEGLAB toolbox was performed (Delorme and Makeig, 2004). Data were then bandpass filtered between 0.01 and 40 Hz and epoched from −100–500 ms. OSL’s artefact rejection algorithm removed residual bad trials and channels. Robust averaging averaged epochs for trials, with conditions separately weighted. A final low-pass filter corrected for potential high frequencies introduced during robust averaging.

### Sensor level analysis

For each participant with mild cognitive impairment or Alzheimer’s disease from the included NTAD participants, data from combined planar gradiometers to the repeated tones (tones 2–11) were subtracted from the first (deviant) tone, giving mismatch negativity waveforms for each participant for each session (baseline and follow-up recordings). Based on prior studies of the mismatch negativity, the average amplitudes between 140–160 ms was calculated for each waveform [27, 28], for each participant, at each session. Two participants were excluded from further sensor level analyses as their mean mismatch negativity amplitudes were three scaled median absolute deviations from the median at both baseline and follow-up sessions [81]. One outlier had a low MMSE of 18, the second outlier had a high MMSE of 27 and both outliers had low mismatch negativity amplitudes. We include in the supplementary data plots showing these outliers’ mismatch negativity amplitudes with the rest of the group. The average amplitude across waveforms was calculated for each participant.

A paired t-test was used to assess change in average amplitude between baseline and follow-up MEG scans. A linear regression was fitted to the average amplitude and baseline MMSE scores.

### First-level dynamic causal modelling

We developed a variant of the conductance-based canonical microcircuit model (cmm_NMDA) in SPM12 version 7771. The standard cmm_NMDA model has spiny stellate, superficial pyramidal, inhibitory interneurons, and deep pyramidal cells in three layers of the cortical column as shown in Fig. 2a. The dynamics of each neuronal population are governed by a Morris–Lecar model, which can be thought of as a reduction of the Hodgkin and Huxley’s squid axon model [82] as follows:

$$\frac{{dV}}{{dt}}= \,	{\frac{1}{C}}[{g}_{L}\left({V}_{L}-V\right)+{g}_{{AMPA}}\left({V}_{{AMPA}}-V\right)+{g}_{{GABA}}\left({V}_{{GABA}}-V\right)\\ 	 +{g}_{{NMDA}}m\left(V\right)\left({V}_{{NMDA}}-V\right)]+u,\frac{d{g}_{* }}{{dt}}=\frac{1}{{\tau }_{* }}\left({\sum}_{k={sp},{inh},{dp},{ss}}{S}_{k}{\sigma }_{k}-{g}_{* }\right)\\ 	 +u,* =[{L}_{c},{AMPA},{GABA},{NMDA}]$$

In Eq. 1, $V$ is the membrane potential; ${g}_{* }(L,{NMDA},{AMPA},{GABA})$, the conductance of ion channels; *u*, thalamic input given by a hump shape function; *C* is the membrane capacitance; ${L}_{c}$ is a passive leak current, ${\tau }_{* }$ are time constants for the ion channels, ${V}_{* }$ are the reversal equilibrium potential of the ion channels. ${\sigma }_{k}$ is the afferent presynaptic firings from a population $k$, which is scaled by ${S}_{k}$, intrinsic and extrinsic connectivity.

Each region can interact with distal regions via forward connections (from superficial pyramidal cells to spiny stellate and deep pyramidal cells) and backward connections (from deep pyramidal cells to superficial pyramidal and inhibitory interneurones).

NMDA channels are both ligand-gated and voltage-gated, requiring both the binding of glutamate and removal of the magnesium blockade by a large transmembrane potential to open. The removal of the magnesium blockade is given by the function:

$$m\left(V\right)=\frac{1.5}{1+0.33\exp (-0.06V)}$$

We adapted the model by including a parameter for NMDA channel blockade (blk_NMDA_) able to vary during model inversion as follows:

$${\alpha }_{{NMDA}}=\exp ({bl}{k}_{{nmda}})$$

$$m\left(V\right)=\frac{1.50265}{1+0.33\exp (-0.06{\alpha }_{{NMDA}}V)}$$

There is a single NMDA channel block parameter for each region in the model representing the blockade of all NMDA channels in the region.

We included left and right inferior parietal cortices from the mismatch negativity network [27, 37, 38]. These are part of a wider network with sources including primary auditory, superior temporal and inferior parietal cortices [83]. However, given the complexity of the neuronal models and their estimation, we only consider the inferior parietal cortex, given its importance in Alzheimer’s disease [43, 84] and mismatch negativity generation. This reduces the complexity of the model inversion [22] and offers a more parsimonious network for investigating NMDA blockade dysfunction. Each of the two regions receive thalamic input and have self-connections that are altered by repetition (deviant versus repetition 5) [85]. There is no lateral connection between the parietal cortices. This minimal network allows us to investigate the dysfunctions of NMDA channel blockade in Alzheimer’s disease.

**Fig. 4:**
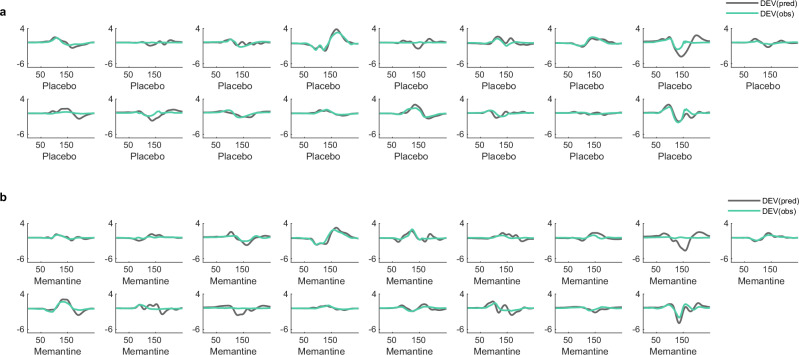
Model fits of the deviant tone for healthy controls from the memantine-placebo study. Observed responses (cyan) and model-generated responses (grey) to the deviant tone of the mismatch negativity paradigm for each participant on (**a**) placebo or (**b**) memantine. DEV, deviant; pred, predicted; obs, observed.

**Fig. 5:**
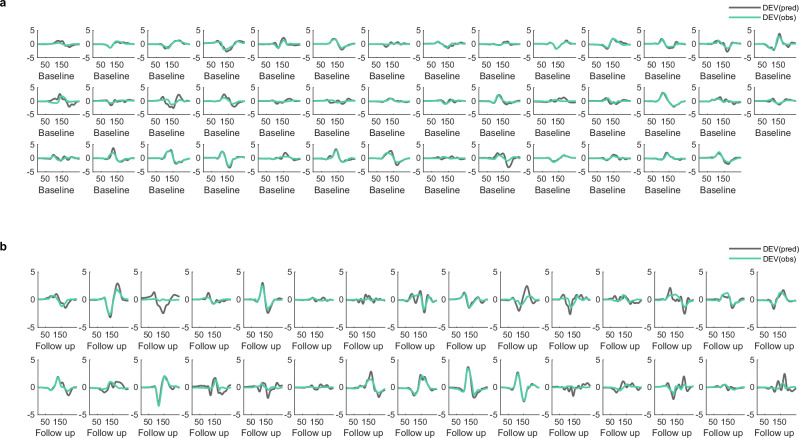
Model fits of the deviant tone for people with Alzheimer’s disease. Observed responses (cyan) and model-generated responses (grey) to the deviant tone of the mismatch negativity paradigm for each participant at (**a**) baseline or (**b**) follow-up session. DEV, deviant; pred, predicted; obs, observed.

The model was inverted from evoked responses to the first and sixth tones (deviant and repetition 5) from the mismatch negativity paradigm for each participant at each session. The between-trial effects (specified in DCM.xU.X) were modelled as 1 for the deviant evoked response and 0 for the standard evoked response (repetition 5). The sensor data were reduced to eight spatial modes from an epoch of 0–300 ms post-stimulus onset and a Hanning window applied. Source activity was approximated during DCM fitting as equivalent current dipoles with symmetry constraints. This method has previously been applied to a simplified (reduced number of sources) DCM network to generate mismatch negativity responses [22]. Subject-specific T1-weighted images informed the lead field. The model is fitted by iterative updating of parameters to improve the fit of the model’s generated response to the observed response, with a trade-off of complexity and accuracy until the parameters maximise free energy [17]. This furnishes a unique set of model parameters for each participant at each session which can be compared with second-level analyses (see Fig. 4 and Fig. 5 for model fits).

### Second-level analysis

We used a hierarchical regression model, namely PEB, that takes both posterior estimates and covariances of parameters to the second level [44]. At the first level of the PEB, DCMs explain how neural activity causes individual evoked responses with all parameters fixed apart from the parameters of interest (see below). The parameters of interest are modelled at the second level with a general linear model. The second level uses a hierarchical variational Bayesian inversion that constrains posterior parameter estimates by the user-specified regressors to improve model evidence. Hypotheses can be tested by comparing the free energy of PEB models with different combinations of parameters explaining the second-level effect.

### Second-level analysis for memantine versus placebo in control participants

Second-level group PEB analyses were performed with different sets of parameters to test biological hypotheses about the effect of memantine on the cortical microcircuit. These parameter sets were: 1) GABA time constants, 2) AMPA time constants, 3) NMDA time constants and 4) NMDA channel blockade. Separate models were run in which only one of these parameter sets was free to vary, plus a fifth model in which they were all allowed to vary. Each PEB included a constant term and a regressor capturing group (memantine versus placebo). The free energies of the PEB models were converted to posterior probabilities with the softmax function to identify the likely PEB model whose parameters best explained the effect of memantine.

### Second-level analysis of NMDA channel blockade in alzheimer’s disease

To explore the pathological effect of Alzheimer’s disease, second level PEB analyses were tested with the winning NMDA channel blockade parameters in the Alzheimer’s disease group. A PEB model was tested in which the mean-centred MMSE baseline values were added as a regressor (along with the constant term). The PEB posterior estimate of the expected value of NMDA channel blockade parameters that differed from 0 with a posterior probability >0.95 were extracted from baseline DCMs for each person with Alzheimer’s disease and plotted against their MMSE score (Fig. 3).

Finally, a longitudinal PEB analysis was also performed on DCMs from people with Alzheimer’s disease, to assess how NMDA channel blockade changes with disease progression, with constant and session (baseline versus follow up) as regressors. A third regressor specified the time in years between the baseline and follow-up scans for each patient (0 for baseline scans and with scores from 0.8–2.2 for follow-up scans mean-centred and standardised). PEB posterior estimates of the NMDA blockade parameter that differed from 0 with a posterior probability >0.95 were extracted and plotted against session.

For each PEB model, Bayesian model comparison and averaging was performed over a model space of all parameter combinations.

## Supplementary information

Supplementary material

## Data Availability

The code associated with this paper is available at https://github.com/jlansk/NMDA_blockade/. Anonymised (unlinked) raw data will be made available via Dementias Platform UK, subject to managed access conditions that protect participant confidentiality and conditions of consent.
