# Supplementary material for: Alzheimer’s disease and memantine effects on NMDA-receptor blockade: non-invasive in vivo insights from magnetoencephalography
Source: Mol Psychiatry. 2025 Oct 10;31(3):1587–96. doi: 10.1038/s41380-025-03288-3 (PMC12916295; doi:10.1038/s41380-025-03288-3)
Supplement: Supplementary file 1 — Supplementary material [file 41380_2025_3288_MOESM1_ESM.docx]

**Supplementary materials**

**Supplementary Methods**

**Supplementary analysis of people with Alzheimer’s disease versus healthy controls**

Demographics of the healthy control participants from the NTAD study are detailed in **Supplementary Table 1**.

| Sex (Male:Female) | Handedness (Right:Left:Both) | Age (yrs)* | Education (yrs) | Baseline MMSE* | Baseline PET | Baseline CSF |
| --- | --- | --- | --- | --- | --- | --- |
| 9:5 | 11:3:0 | 65.3 (±7.29) | 16.2 (±3.38) | 29.4 (±0.74) | 0.98 (± 0) | 0.38 (±0.21) |

**Supplementary Table 1 NTAD control participant demographics**

DCMs were inverted in line with the main analyses.

A PEB analysis was run with a design matrix that comprised of a regressor of ones, a second regressor for group (0 for controls, 1 for patients) and a third regressor to control for age ( mean centred and scaled). PEB posterior estimates of the NMDA blockade parameter were extracted and plotted against age.

**Fitting first-level DCMs**

There were two inversion failures when fitting DCMs to the data from the memantine session of the memantine-placebo study. There was one inversion failure when fitting DCMs to the patient NTAD data. The respective participants were excluded from the PEB analyses.

**Including age as a covariate in the PEB analysis with MMSE**

We reran the PEB analysis investigating the effect of MMSE on NMDA parameter blockade controlling for age (with a third regressor with centred and scaled age of each participant). The effect of MMSE on the NMDA blockade parameters was unchanged when controlling for age. NMDA channel blockade was reduced with more severe Alzheimer’s disease (a lower MMSE score) with a meaningful effect (probability of parameter > 95%) in right parietal cortex (posterior estimate = 0.06, posterior probability = 0.99).

**PEB analysis of participants with Alzheimer’s disease versus control participants**

NMDA channel blockade was higher in patients than controls with a posterior probability > 95% in left (posterior estimate = 0.51, posterior probability = 1) and right (posterior estimate = 0.28, posterior probability = 1) parietal cortices. Age also increased NMDA channel blockade (posterior estimate = 0.17, posterior probability = 1) in the left parietal cortex (see **Supplementary Figure 9**).

**Supplementary Results**

**
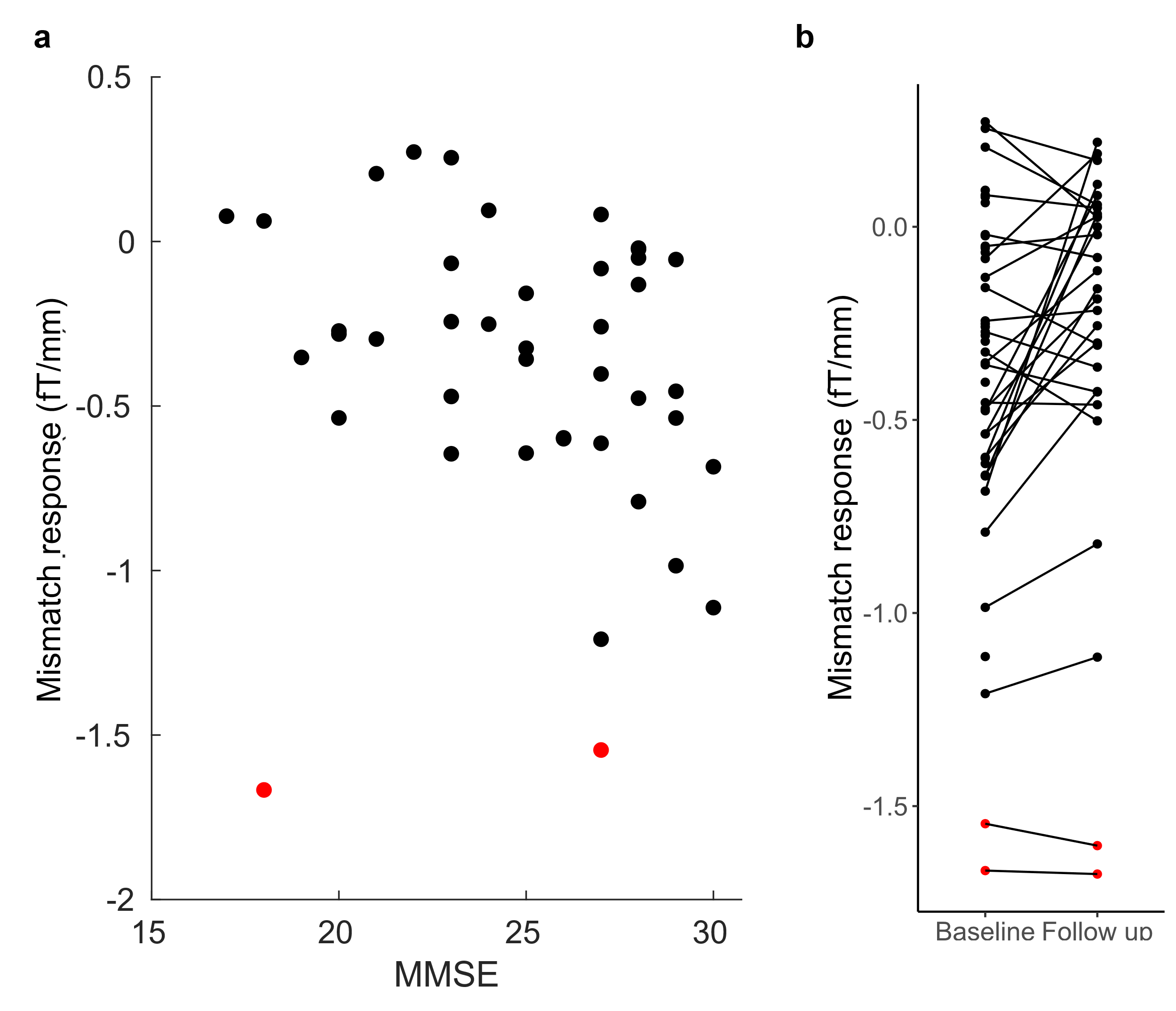
**

**Supplementary Figure 1 Outliers excluded from sensor level analyses**

Scatter plots showing mismatch negativity amplitude for people with Alzheimer’s disease, including the two outlier participants (in red) who were excluded from the sensor statistics against a) MMSE and b) session (baseline versus follow up)


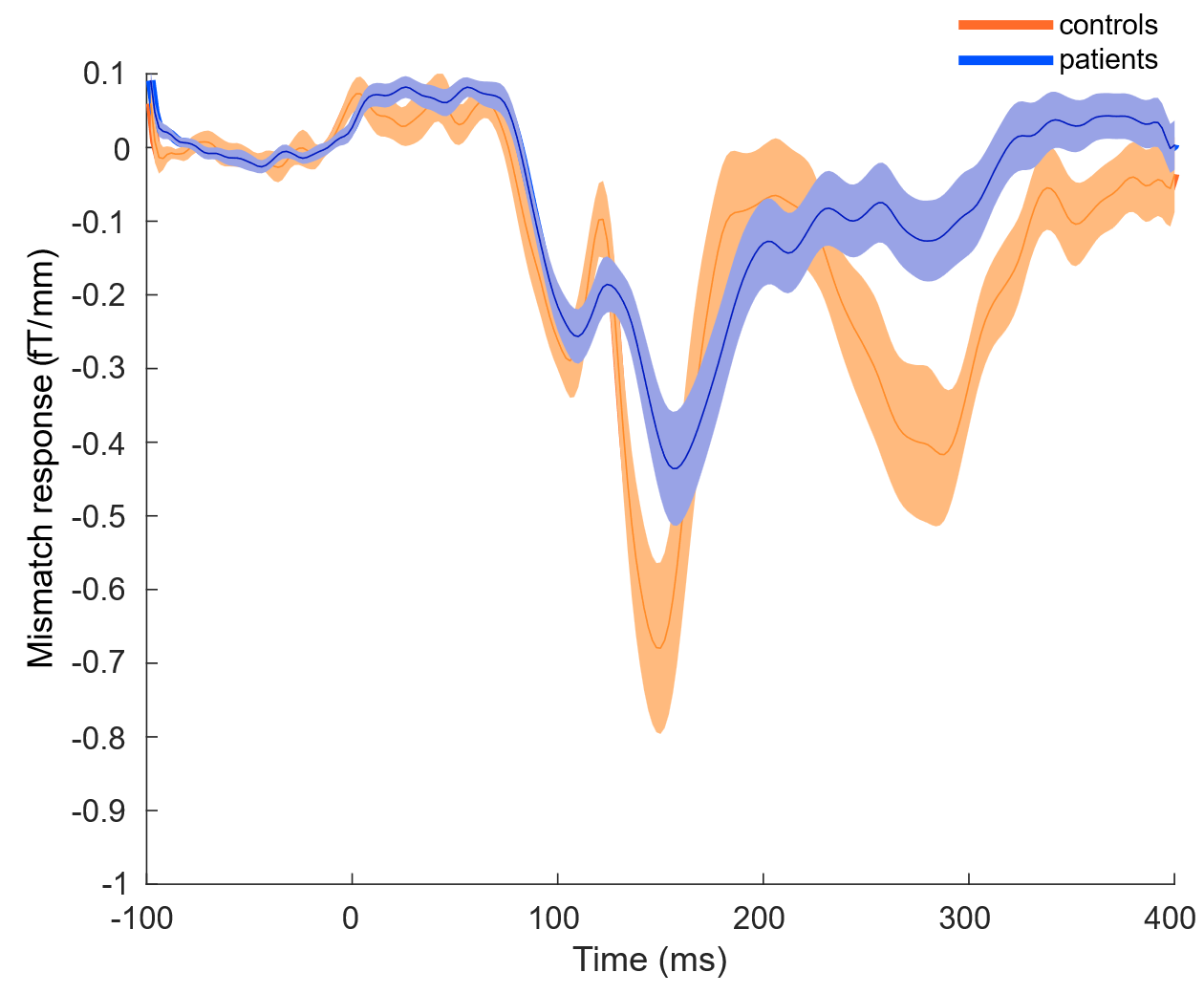


**Supplementary Figure 2 The mismatch response for NTAD participants at baseline.**

The amplitude from 140 to 160ms was significantly reduced in patients compared to controls at baseline (t = -2.56, p = 0.007) with a medium effect size (d = 0.76).


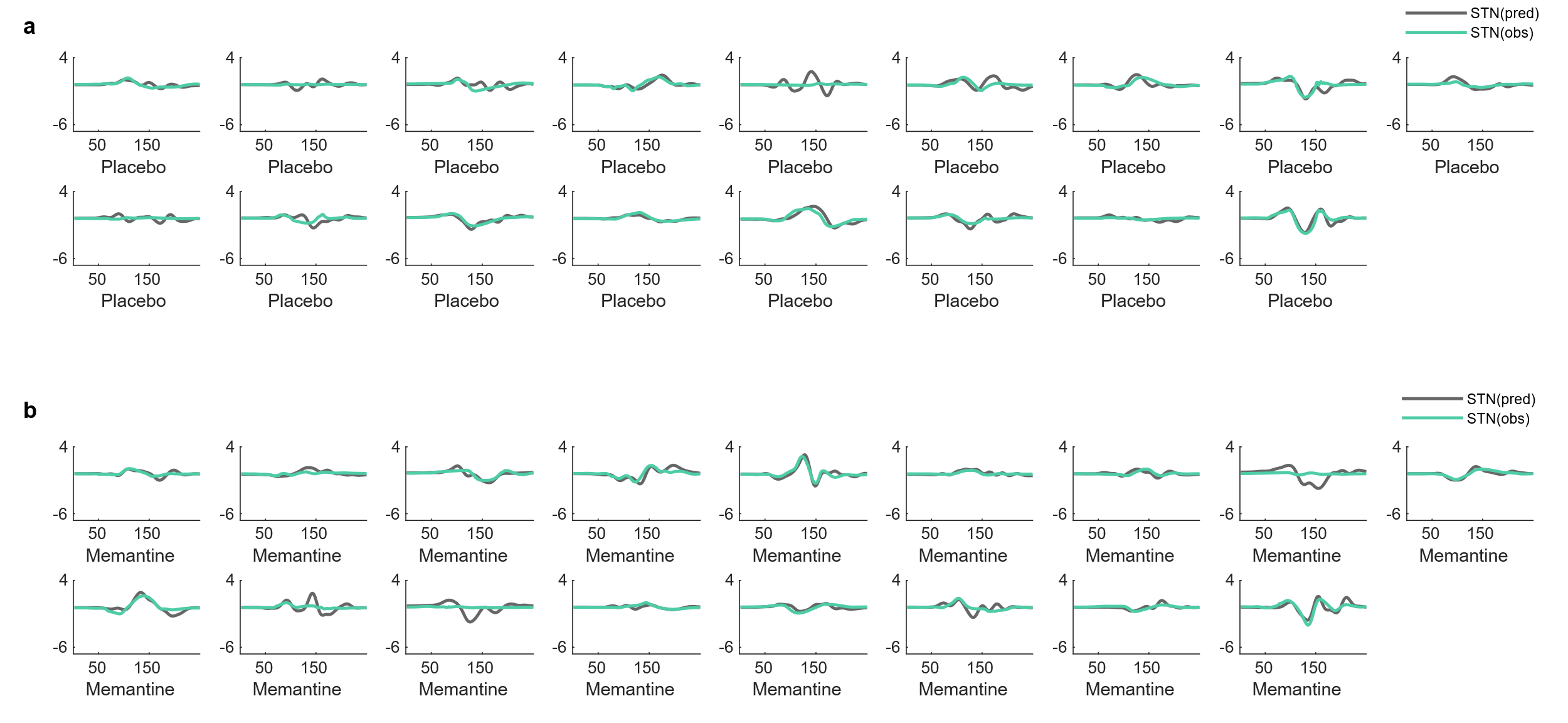

**Supplementary Figure 3 Model fits of the standard tone for healthy controls from the memantine-placebo study.** Observed responses (cyan) and model-generated responses (grey) to the standard (repetition 5) tone of the mismatch negativity paradigm for each participant on a) placebo or b) memantine.

**
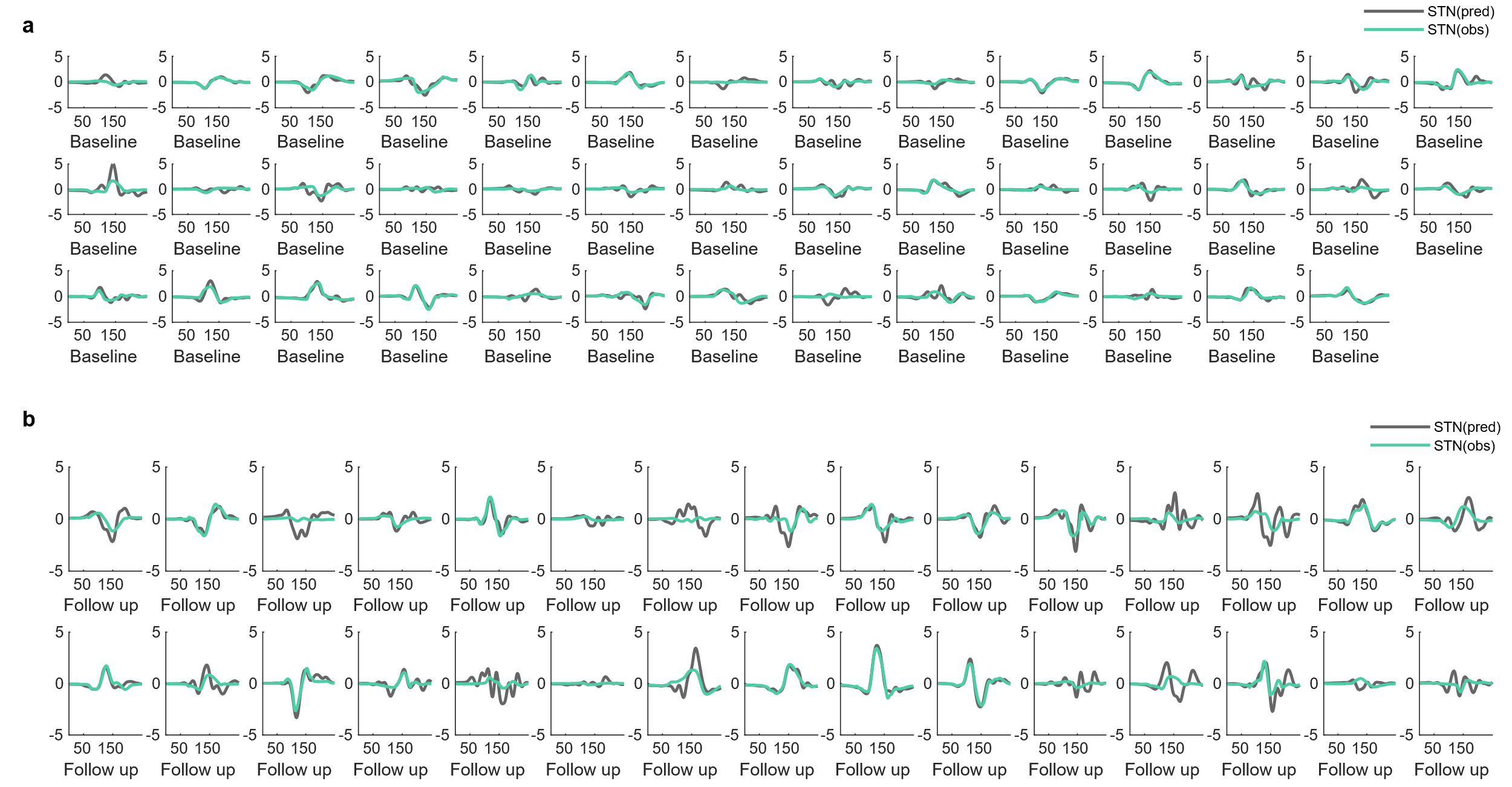
Supplementary Figure 4 Model fits of the standard tone for people with Alzheimer’s disease.** Observed responses (cyan) and model-generated responses (grey) to the standard tone (repetition 5) of the mismatch negativity paradigm for each participant at a) baseline or b) follow-up session.


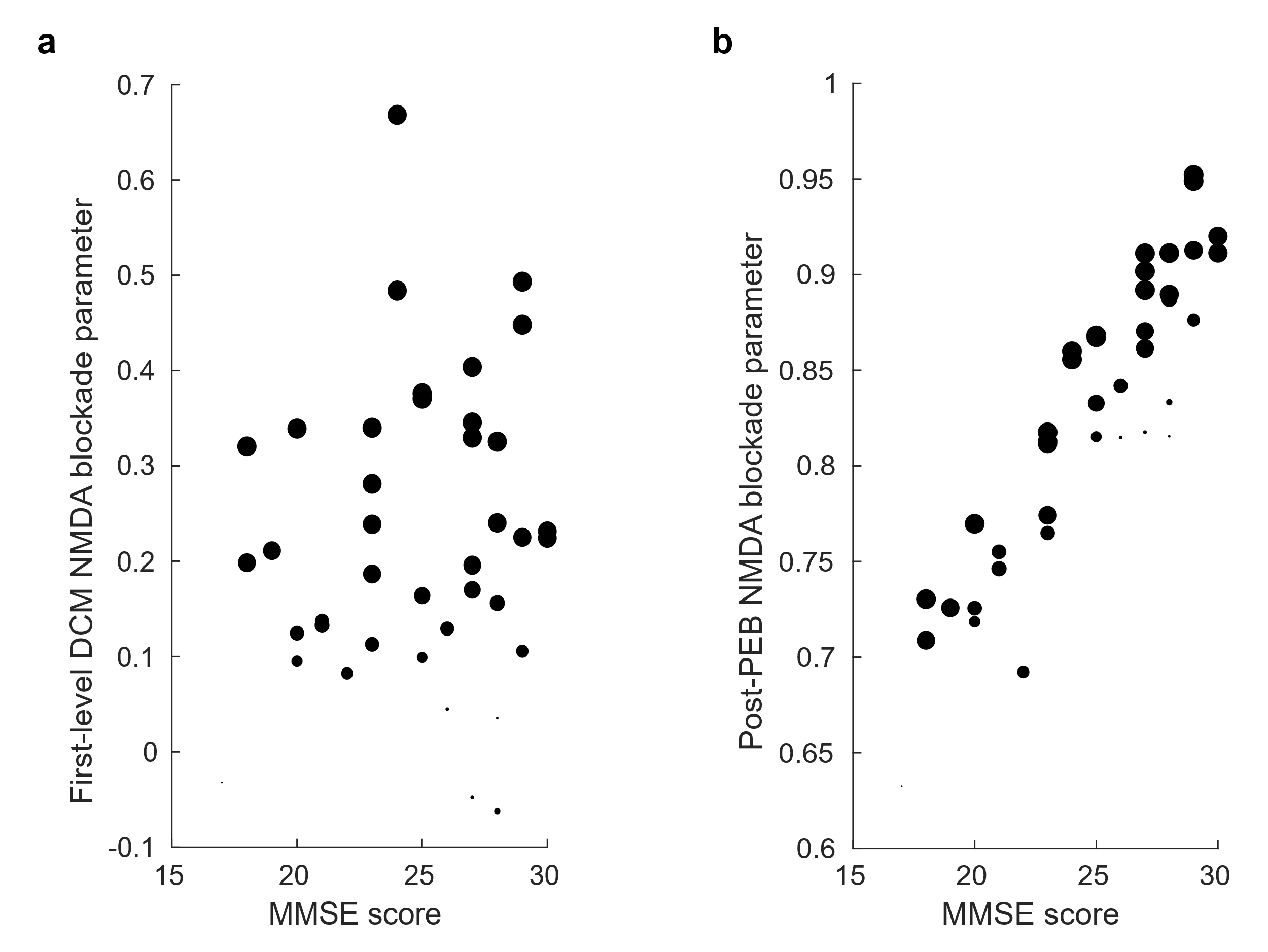
**Supplementary Figure 5 MMSE scores and DCM NMDA receptor blockade parameters** weighted by posterior probability from (a) first-level DCMs weighted by precision (b) second-level (post-PEB) DCMs.


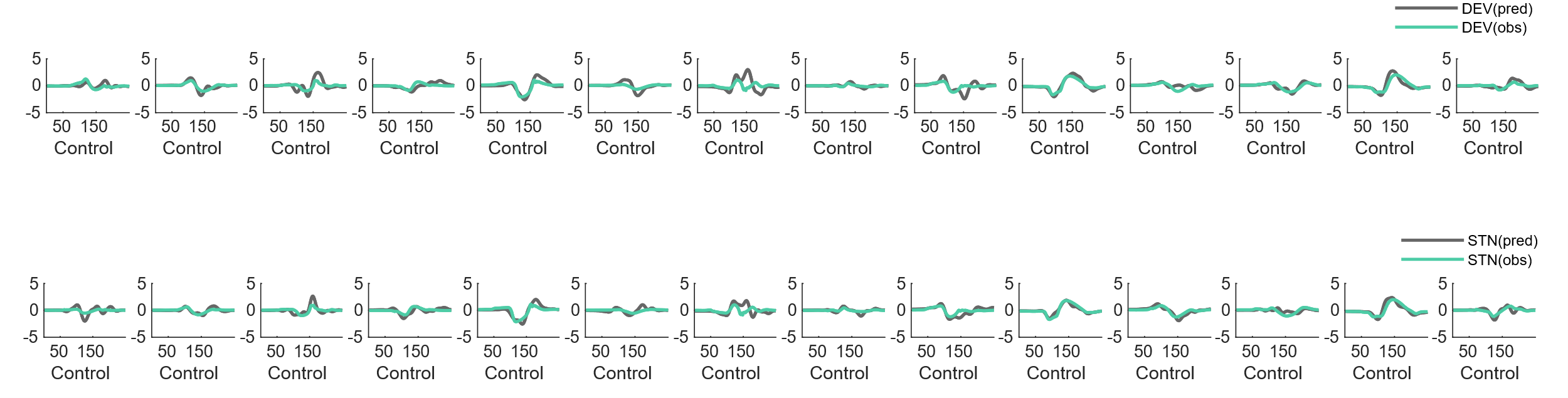


**Supplementary Figure 6 Model fits for the healthy controls from the NTAD study**


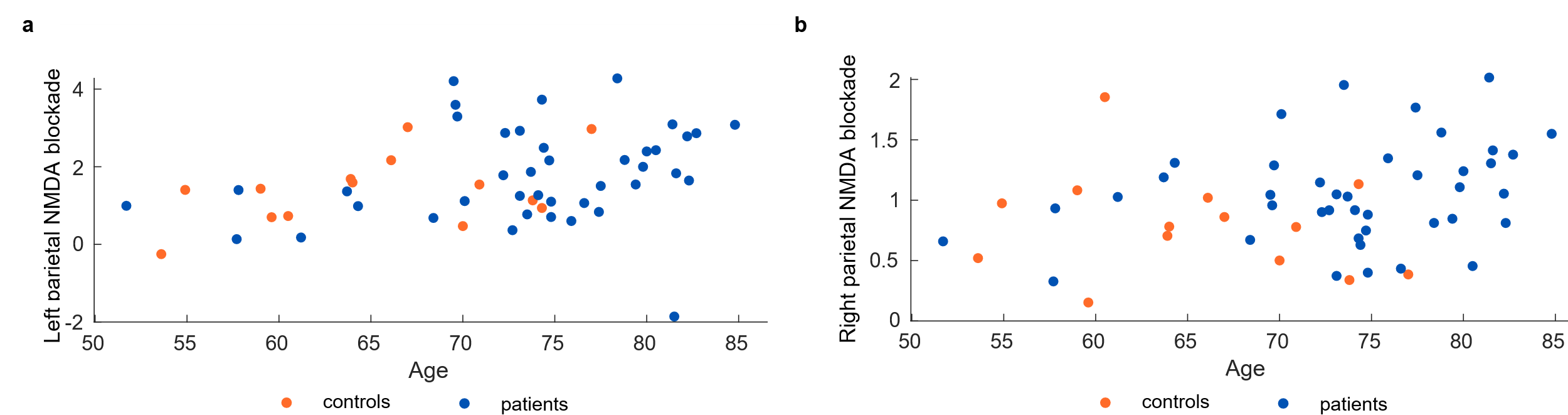


**Supplementary Figure 7 The effect of age and group on the NMDA blockade parameter**

The PEB posterior estimate of the expected value of the NMDA blockade parameter plotted for controls and patients for the **a)** left parietal region and **b)** right parietal region
